# Medical and Philosophical Intelligence

**Published:** 1818-08

**Authors:** 


### MEDICAL AND PHILOSOPHICAL INTELLIGENCE.

*PROCEEDINGS of the Royal Society.* *June* 4.—A description of the teeth of the Delphinus Gangeticus was presented to the Society by Sir EVERARD HOME, Bart. V. P. R. S. And at the same meeting, Dr. GRANVILLE gave an account of the production of sulphuretted azote in the abdomen, resulting from the decomposition of an albuminous dropsical fluid. The Doctor considers this as a new and definite gaseous compound, and the results of his experiments led him to consider its component parts as

89.60 azote.

10.40 sulphur.

A Paper was also read by JOHN WILLIAMS, Esq. describing the influence of galvanism upon the germination of seeds, which, when powerful enough to do any thing, appeared generally injurious.

*June* 11.—Dr. PROUT communicated a Paper, describing a new acid principle, prepared from the lithic or uric acid.

Our readers are well aware of the characteristic property of uric acid of producing a fine red compound, when heated with nitric acid.

Dr. PROUT shows, by some very interesting experiments, that this is a compound of a new acid principle with ammonia. This acid forms purple or red compounds, with the metallic oxides, whence he calls it the *purpuric acid.*

A communication was also received from Sir W. HERSCHEL, consisting of astronomical observations and experiments, selected for the purpose of ascertaining the relative distances of clusters of stars, and of investigating how far the power of our telescopes may be expected to reach into space, when directed to ambiguous celestial objects.

The President then adjourned the Society for the long vacation, which terminates on the 5th of November.

---

A Meeting of the *Associated Apothecaries and Surgeon-Apothecaries* was held on the 15th July, agreeably to the 19th Resolution of the general meeting, in August 1817. After stating the general object of the respective Bills brought before Parliament by the College of Surgeons and Society of Apothecaries, and the advantages that would be derived from them, the Report states, that this Committee—" Determined to take no steps which might prevent the passing of a Bill which, when modified, would have produced so much good, have carefully avoided any impeding interference, declared their general approbation of the Bill, and waited its going into a committee, to suggest such regulations as might appear to be necessary. But they cannot avoid expressing their regret that the Bill was not allowed to go into a Committee of the House of Commons, where its merits might have been appreciated, and such alterations introduced, as would have secured the interests and welfare of the public, so far as concerns the practice of surgery and midwifery.

" Fully sensible of the importance of the general objects of that Bill, they ardently hope that the period is not far distant when the subject will be taken into serious consideration by Parliament itself.

" The measure which the committee must first suggest, regards the duly organizing of the Association into a Society, bearing an appropriate designation, and regulated by fixed laws. The necessity of this must appear, when it is considered, that at present the association is almost without a defined object; that its rules and regulations are wholly incomplete, and scattered through the numerous resolutions, passed at various meetings of the Committee and of the Association. In fact, as its laws consist solely of certain resolutions hastily formed on various occasions to meet emergencies as they have arisen, your Committee thinks it right, earnestly to recommend to the general meeting, that ***steps be immediately taken, to give to this Association a determinate name, form, and object***; and that a committee be appointed to embody the existing resolutions into a code of rules and regulations for the government of the Society; and, as soon afterwards as may be, to lay the result before a special general meeting, to be convened for this purpose; making therein such alterations and improvements, as shall appear necessary.

"Whilst thus modelling the Society, the Committee would make those arrangements, by which committees should be formed in the different counties of England and Wales. The business of these committees would be to watch over the operation of the 'Act for better regulating the Practice of Apothecaries throughout England and Wales,' in their respective neighbourhoods; and to correspond with the parent society, transmitting to them such facts, duly authenticated, as may furnish the legislature with the means of fairly judging, as to the mischief arising from the unrestrained admission of ignorant and uneducated men to trifle with the healths and lives of their fellow-creatures.

---

*Moirè Metallique, or Fer blanc moirè.*—This is an article of Parisian manufacture, much employed to cover ornamental cabinet work, dressing boxes, telescopes, opera glasses, &c. &c. and is prepared in the following manner.

Sulphuric acid is to be diluted with seven or nine parts of water, then dip a sponge or rag into it, and wash with it the surface of a sheet of tin, which speedily will exhibit an appearance of crystallization, which is the Moiré.*

This effect, however, cannot be easily produced upon every sort of sheet tin, for, if the sheet has been much hardened by hammering or rolling, then the moiré cannot be effected until the sheet of tin has been heated so as to produce an incipient fusion on the surface, after which the acid will act upon it and produce the moiré. Almost any acid will do as well as the sulphuric, and it is said that the citric acid, dissolved in a sufficient quantity of water, answers better than any other.

The moiré has of late been much improved by employing the blow-pipe, to form small and beautiful specks on the surface of the tin, previous to the application of acid.

When the moiré has been formed, the plate is to be varnished and polished, the varnish being tinted with any glazing colour, and thus the red, blue, green, yellow, and pearl-coloured, moirés are manufactured.

---

* The word Moiré signifies *watered*, as La Soie Moirée, watered silk.

*Meteoric Iron.*—There is a character first pointed out in Germany, belonging to meteoric iron, which is, perhaps, not very generally known. It consists in the production of regular figures and crystalline facets on the polished surface of the iron, when moistened with nitric acid, analogous to those produced in the moiré metallique. This character has been found to belong to all the well known specimens of meteoric iron that have been tried, and as distinctly in the grains found in meteoric stones, as in larger masses of the metal; but it has been looked for in vain in the native iron of Charlesdorf, of Veiben, of the hill of Briandi (de Chladni,) of Peru, and in the mass at the Cape, first made known by Barrow and Dankelmann.

---

*Reduction of Chloride of Silver by Hydrogen.*—The following method of reducing chloride of silver is perhaps not sufficiently known. It was communicated by M. Arfwedson. Liberate hydrogen in contact with chloride of silver, as by mixing the chloride, zinc, sulphuric acid, and water together, and the silver will be reduced to the metallic state; the zinc is easily dissolved out by excess of acid, and the metal obtained by filtration or decantation.

---

*Oxide of Lead crystallized*—Mr. Houtan Labillardiere has described a crystallization of the oxide of lead from its solution in soda. The solution had been left to itself during the winter, and had deposited many small crystals of a regular dodecahedral form, white, and semi-transparent. They were insoluble in water; on burning charcoal, were reduced to lead; and were not diminished in weight when heated in a platinum crucible. They dissolved without effervescence in nitric acid; the solution was not rendered turbid by the nitrates of silver or barytes, but threw down a black precipitate with the hydrosulphuret of potash, and a white one with sulphuric acid, which was insoluble in nitric acid. These results prove the crystals to be pure oxide of lead.

---

*Crystallized Iodine.*—Some curious observations on the forms of crystallized iodine have been published in the Bibliothèque Universelle. Crystals had formed on the surface and at the bottom of a solution of iodine, by slow evaporation, and were all of them cubes. In another solution they had formed in great abundance on the surface, and in the upper part of the bottles; and with the exception of a single crystal, which was rhomboidal, were perfect cubes; some of them were as much as half a line in the side. The crystals increased rapidly in size, although the temperature of the place was never above 45.5° of Fahrenheit, and was frequently at the freezing point of water.

In the distillation of iodine from water, very fine crystals were obtained, but of a different form. The apparatus was a retort, an adapter, and a globe. The adapter was preserved at a low temperature, and soon became lined with crystals resembling fern leaves. Each leaf appeared to be attached to the glass by a sort of stalk, and they affected a parallelism with the axis of the adapter. Some of them were nearly an inch in length, diminishing gradually in width towards the end.

---

*New Products from Coal.*—It is said that Dr. JASSMEYER, Professor of Chemistry in Vienna, has made the discovery of a means to extract from coals two hitherto unknown acids, a resin, a resinous gum, and other products, which he has employed with success in the dying of wool, silk, hair, and linen; and that he has produced from them red, black, yellow, and various shades of brown and grey. The President of the Aulic Chamber, and other enlightened judges of these matters, have given their approbation to the discovery.

---

*New Metal.*—M. STROMEYER has discovered the existence of a new metal in the ores of zinc; he calls it *cadmium*. It looks like tin, and its chemical habitudes much resemble zinc, but it is precipitated from its solution in the metallic state by the latter metal.

---

*New Mineral—Hydrate of Silica and Alumina.*—M. LEON DUFOUR has found a mineral in the neighbourhood of Saint Sevér, which appears to be new. It occurs in an argillaceous, gravelly soil, in detached pieces, from two to four or five inches in diameter. It is generally of a fine white colour, without lustre, but is found sometimes with the semi-transparency of opal. Its hardness is between that of limestone and lithomarga, and in many characters it approaches to the latter substance. Its fracture is dull; its composition homogeneous. It is easily cut by a knife, and yet is singularly fragile: when struck by a hammer, it breaks into very angular pieces. It is soft to the touch, and may be polished very highly by friction. It adheres strongly to the tongue, but has no argillaceous or earthy odour when breathed upon. It does not effervesce with acids, nor form with water a ductile paste. Its colour is not changed by heat. It has been observed to diffuse a very singular smell of apples, particularly when newly fractured.

An analysis, made by M. Pelletier, has given the constituents of 100 parts of this mineral, as silex 50, alumine 22, water 26, there being a loss of 2 parts.

---

*Siliciferous Subsulphate of Alumine.*—Dr. HENRY, of Manchester, has described and analysed a peculiar substance, apparently the result of slow chemical action, found in the old hollows of a coal-mine. It has exactly the appearance, as well as consistency, of hogs-lard, and was mistaken at first for it by the miners. Its taste is sub-acid. It dries in the air, splitting like starch. When heated strongly, it becomes so hard as to scratch glass. An analysis gave its proportions as follows: water 88.1; alumine 6.5; sulphuric acid 3.0; silica 2.4. It has been called siliciferous subsulphate of alumine.

---

Mr. Alcock advocates the treatment of Syphilis without Mercury, to which he appears originally to have been led by accident. A large neglected chancre in a gentleman (who, from peculiar circumstances, could not employ mercury,) was cured by escharotics, and no secondary symptoms appeared. He has continued the same practice for the last ten years without once having occasion to regret it. Although, in the case related, we may infer from the term "neglected," that it was not of the most recent origin, and was, notwithstanding, cured; yet Mr. A. appears disposed to limit the application of escharotics to the first week of the appearance of chancre. Sometimes, however, he uses them later, but never when there is any perceptible affection of the neighbouring glands. The best remedies for this purpose are sulphate of copper and nitrate of silver,—the pure potash being liable to spread, its use is less proper after the slough, occasioned by the caustic, separates. If the sore has not lost the specific character, it may be repeated two or three times; and, if its operation be favourable, "the sore is increased in size, but it becomes florid, and divested of much of its characteristic hardness, after which it heals rapidly by simple dressing or cleanliness:" sometimes the sores require to be dressed with stimulants. Mr. A. is thoroughly persuaded that primary symptoms can be cured by this treatment in less time than by mercury, and without the supervention of secondary symptoms; and that secondary symptoms (as is evident from many cases already before the public,) frequently wear themselves out; yet, he doubts whether this spontaneous cure is so short or so certain as the treatment of mercury judiciously employed. This practice, he observes, was known to Mr. Hunter, who removed the chancre by incision, and by caustics, when it healed as a common sore; nevertheless, he rendered the conclusion defective by advising mercury to be conjoined with it. To remove all doubt as to the syphilitic nature of the sores treated by the author, he describes the characteristic symptoms of chancre which were present in most of his cases:—"loss of substance; absence of granulations; the matter adhering to the surface of the ulcer; thickened base; abrupt and elevated edge, with circumscribed hardness; and, in its progress, extending in every direction from the point first affected." The necessity of *an early application* of the caustic is urged from a comparison with the practice of excising the wound in the bite of a rabid animal, which it is well known must be resorted to immediately.

---

*On the Chrystalline Form of the Deutoxide of Lead;* by M. Houton la Billardiere. (Abridged from Journ. Pharm. for Aug. 1817.)—The author informs us, that by boiling massicot in a solution of caustic soda, a portion of the metallic oxide is dissolved; and that, after a considerable length of time, the solution deposits white semi-transparent crystals, of the size of a pin's head, which, by means of a microscope, are easily discovered to be regular dodecahedrons. He performed a series of experiments on these crystals in order to ascertain their nature, and he satisfied himself that they consisted of pure oxide of lead. He styles it the *deutoxide*, because he supposes that there is an oxide with a smaller proportion of oxygen, which may be procured by calcining the oxalate of lead; but it is in fact the *protoxide*, or yellow oxide of the systematic writers.

---

*Shower of Red Earth in Italy.*—A shower of red earth fell at Gerace, in Calabria, on March 14, 1813. The wind had been westerly for two days, when at two P. M. it suddenly became calm, the atmosphere grew cloudy, and the darkness gradually became so great as to render it necessary to light candles. The sky assumed the colour of red-hot iron, thunder and lightning continued for a considerable length of time, and the sea was heard to roar, although six miles from the city. Large drops of rain then began to fall, which were of a blood-red colour.

Sig. Sementini describes the physical properties of the powder as follows:—It had a yellow colour, like canella; an earthy, insipid taste; unctuous to the touch, and extremely subtile. When moderately heated it changed its colour, first to a brown, and afterwards to a black, and became red again as the temperature was raised; after it had been heated, many small shining plates were visible; it no longer effervesced with acids, and had lost about $\frac{1}{10}$ of its weight. Its specific gravity was 2·07.

Its composition was,

| | |
|---|---|
| Silex | 33 |
| Alumine | 15½ |
| Lime | 11½ |
| Chrome | 1 |
| Iron | 14½ |
| Carbonic acid | 9 |
| Loss | 15½ |
| | 100 |

So great loss was at first ascribed to some inaccuracy in the analysis, or to some body that had accidentally been mixed with the powder, but as it always occurred whatever care was taken in the analysis, he began to suspect that it depended upon some combustible matter essential to the substance. This suspicion was afterwards verified; and, by digesting the powder in boiling alcohol for a length of time, he obtained from it a greenish-yellow coloured matter, which, when dried, acquired a pitchy consistence,—was inflammable, and left a carbonaceous residuum. The author remarks, that the existence of chrome in this mineral seems to connect it with the aerolites, but the origin of the combustible substance is very obscure; there were no circumstances connected with the phenomenon which would lead us to suppose that it was of volcanic origin.

*Account of a new Mineral called Pargasite.*—A new mineral called pargasite has been sent to this country from Finland. It was found some years ago at the village of Ersby, near Abo.

It is of a green colour, is translucent, and transparent. Its crystals are of various sizes, from an inch downwards. Its form is an octohedron, with a rhomboidal base. It has three cleavages. It is harder than fluor spar, but is scratched by quartz. It also scratches glass. Specific gravity 3·11. It melts before the blow-pipe into a mass of a pearly white lustre. The following are given as the proportion of its constituents:

| | |
|---|---|
| Selix | 42·01 |
| Magnesia | 18·27 |
| Lime | 14·28 |
| Alumine | 14·08 |
| Oxide of iron | 3·52 |
| Oxide of manganese | 1·02 |
| *Oxide of a metal not investigated* | 0·33 |
| Fluoric acid and water | 3·09 |
| Loss | 2·59 |
| | 100·00 |

*Coal Gas employed for the Blow-pipe.*—We are informed that M. Lampadius, on making use of the gas blow-pipe, has found the heat, which is produced by the combustion of oxygen with carburetted hydrogen procured from coal, to be more intense than that with pure hydrogen.—*Journ. Phys. for Jan.* 1818.

*Analysis of a Specimen of the Diamond Rock.*—Dr. E. D. Clarke, Professor of Mineralogy at Cambridge, has examined a specimen of the diamond rock, from the banks of the river Jigitonhônha, in Brazil, which was sent to him for this purpose by Mr. Mawe, of the Strand. It contains diamonds in their matrices. The rock consists of an aggregate of small quartz pebbles firmly set in indurated iron-sand. The cavities in which the diamonds are placed are invested by a yellow ochreous matter, which is full of minute pallets of native gold, visible to the naked eye.

In reference to another subject, the editor remarks, that the metallic lustre, with which pure silica becomes invested when fused before the gas blow-pipe, is sometimes due to the charcoal used as a support; the same appearance takes place, under similar circumstances, in the fusion of corundum, and other refractory bodies, such as magnesia and lime. But Dr. Clarke has observed globules of a metal resembling silver when silica has been fused with borax before the gas blow-pipe, the difficulty of obtaining which is owing to their volatilization almost in the instant of their being formed. A still more remarkable result was obtained in the fusion of apatite, when a globule of metal was obtained, which is now in the possession of Mr. Lunn, of St. John's College, Cambridge. It is not pretended to account for these phenomena, but merely to state the facts, in the hope that others may confirm and explain them.

The *Dictionnaire des Sciences Medicales* contains the following remarks on the use of Belladonna, which seems likely to become a valuable article of the Materia Medica.

Numerous experiments, made during the last thirty years, on belladonna, enable us to appreciate pretty correctly the advantages of this vegetable. Notwithstanding the assertions of M. Muench, and the observation of M. Bucholtz, of Weimar, who assures us that he cured a case of hydrophobia, that had already unequivocally declared itself, by belladonna, we are constrained to reject all prospect of success from this remedy in rabies canina. Its inefficacy in cancerous affections has been demonstrated by many testimonies, but particularly by the experiments of M. Rahn, of Zurich. It appears to have been successfully employed as an anti-syphilitic in chronic cases unattended by inflammation. M. Boettcher, of Konigsberg, exhibited a composition of belladonna and calomel in phagedenic ulcers of the throat and of the genital organs with marked and rapid benefit. But it is in the class *neuroses* that belladonna has evinced its greatest power, especially in epilepsy and mania. J. E. Greding, physician to the Pauper Establishment at Waldheim, has been long employed in ascertaining the effects of belladonna in these diseases; and his experiments, published by his son in 1790, are interesting. Greding exhibited the powder of the plant, in cases of epilepsy, in doses from half a grain to a grain and a-half, three or four times a-day; also a combination of powder and extract (three parts of extract to one of powder) in doses from three to ten grains in the twenty-four hours. But, notwithstanding the apparent safety with which M. Greding administered the remedy, in these cases, we think the dose too large, since it occasioned in a young man the loss of vision for the space of twenty-one days. Administered to twenty-three maniacs, the belladonna did not effect a perfect cure in any one; but the force of the paroxysms was greatly moderated, and the morbid symptoms unequivocally ameliorated.

"I have myself (says Marc) employed the extract of belladonna externally, and with some success, in tic douloureux. A physician of my acquaintance assures me that he has cured an obstinate and chronic neuralgia affection of the face by the internal administration of this vegetable." There are few medicines whose utility in hooping-cough has been more decidedly proved than belladonna, and yet it is surprising that medical men should be so negligent in making use of it. M. Schaeffer administered this remedy to infants of one, two, and three years of age, every two hours, with great advantage. Several physicians, among whom we may cite M. Hufeland himself, have, since that period, tried M. Schaeffer's method, and all unanimously agree that belladonna may be considered as almost a specific in hooping-cough. This also accords with my own experience in three most obstinate cases of the disease. At this moment, I am giving the medicine to two young children afflicted with hooping-cough, and with decided advantage. During the epidemic prevalence of this distressing disease at Augsburg, in

1810, M. Wetzler treated thirty children by belladonna, and cured them all in a space of time varying from eight to fifteen days from the commencement of the remedy. M. Wetzler gave the root of belladonna in powder, mixed with a little sugar, in doses of a quarter of a grain night and morning, to children under twelve months, and in relative proportions as the ages increased.

The simplicity of this mode of treatment, its facility of application, even among the most indigent classes of society, and the little repugnance which children manifest towards the remedy, are all advantages which entitle belladonna to a more extended trial than it has yet obtained.

---

Mr. Gaulter will deliver his Course of Lectures on Physiology in October next.

---

### REPORT OF DISEASES.

From about the twentieth to the twenty-sixth of the preceding month the heat of the weather has been singularly oppressive. Since our last report, fevers have been generally on the increase; of these by far the greater proportion have been accompanied by marked disorder of the chylopoietic viscera, and have been received under the denomination of bilious complaints. Typhus fever has also been prevalent, and some cases of both species have resisted both the powers of nature and the efforts of art, and have terminated fatally. The characteristics of the bilious fever have been clear and defined. Head-ach was its general precursor, with lassitude and general indisposition. The tongue was considerably furred, and the febrile exacerbations occurred at noon as well as in the evening. In some instances abscesses took place at the termination of the febrile excitement; and in one, death will probably ensue in consequence of the exhaustion produced by an extensive collection of matter in the vicinity of the ham.

Many cases of head-ach from plethora have been observed, which yielded to bleeding in some of its forms, local or general. The nitrate of potash, with purgatives, were had recourse to as an adjuvant, and in some of the lighter forms of the disease, were evidently serviceable in lessening the arterial action, which formed the prominent feature of the complaint.

Children seem also to have partaken of this increased vascular excitement, convulsions having been very common from this source; but the application of leeches to the temples has in every instance been successful.

Stramonium has been found useful in cases of chronic rheumatism, in the dose of half a grain of the powder three times a-day. This form of administering the remedy is infinitely preferable to that of the extract, being more certain in its operation.

### METEOROLOGICAL JOURNAL,

By Messrs. WILLIAM HARRIS and Co. 50, Holborn, London.

*From the 20th June to the 19th July, inclusive.*

| Day of Month. | Moon. | Rain gauge | Therm. | | | Barom. | | De Luc's Hygrom. Dry | Damp | | Wind. | | Atmospheric Variation. | | |
|---|---|---|---|---|---|---|---|---|---|---|---|---|---|---|---|
| June | | | | | | | | | | | | | | | |
| 20 | | | 61 | 67 | 51 | 29·66 | 29·85 | | 10 | 12 | WSW | WSW | Fine | Rain | Fine |
| 21 | | ,07 | 54 | 62 | 50 | 29·92 | 29·91 | | 14 | 14 | SW | SW | Fine | Clo. | |
| 22 | | | 57 | 64 | 50 | 29·59 | 29·61 | | 16 | 10 | SW | SW | Rain | | |
| 23 | | ,10 | 60 | 65 | 59 | 29·80 | 29·86 | | 2 | 7 | SW | SW | Fine | | |
| 24 | | | 62 | 70 | 64 | 30·30 | 30·41 | | 11 | 9 | NW | SW | Fine | | |
| 25 | ☽ | | 67 | 75 | 65 | 30·12 | 29·99 | | 6 | 6 | SW | WSW | Clo. | Fine | |
| 26 | | | 67 | 76 | 66 | 30·00 | 30·03 | | 5 | 7 | SW | SSW | Fine | | |
| 27 | | | 69 | 79 | 62 | 30·00 | 29·73 | | 6 | 5 | WSW | SW | Fine | | |
| 28 | | | 67 | 78 | 58 | 29·79 | 30·00 | | 5 | 5 | SSW | SW | Fine | | |
| 29 | | | 65 | 80 | 60 | 30 00 | 30·00 | | 6 | 5 | SW | SW | Fine | | |
| 30 | | ,04 | 69 | 79 | 60 | 30·21 | 30·18 | | 7 | 3 | SW | WSW | Fine | | |
| July | | | | | | | | | | | | | | | |
| 1 | ● | | 65 | 77 | 60 | 30·12 | 30·03 | | 3 | 10 | W | NW | Fine | | Rain |
| 2 | | | 63 | 70 | 58 | 30·10 | 30·13 | | 10 | 7 | NE | SE | Fine | | |
| 3 | | | 63 | 69 | 60 | 30·09 | 30·03 | | 8 | 9 | NW | NW | Fine | | |
| 4 | | | 67 | 71 | 63 | 30·03 | 30·05 | | 10 | 7 | NW | NW | Fine | | |
| 5 | | | 69 | 79 | 65 | 30·01 | 30·05 | | 8 | 6 | NW | WNW | Fine | | |
| 6 | | | 71 | 81 | 62 | 30·00 | 30·07 | | 3 | 3 | W | SSE | Fine | | |
| 7 | | | 67 | 78 | 58 | 30·06 | 30·00 | | 5 | 4 | SE | W | Fine | | |
| 8 | | | 61 | 71 | 60 | 30·00 | 30·01 | 0 | 0 | 2 | WNW | NW | Fine | | |
| 9 | | | 65 | 73 | 60 | 30·03 | 30·10 | | 7 | 3 | NNW | NW | Fine | | |
| 10 | | | 67 | 76 | 65 | 30·00 | 30·00 | | 5 | 5 | N | SW | Fine | Clo. | |
| 11 | ○ | | 69 | 75 | 67 | 29·98 | 29·81 | | 7 | 9 | N | W | Fine | | Rain |
| 12 | | | 68 | 77 | 65 | 29·78 | 29·77 | | 11 | 12 | SW | W | Rain | Clo. | |
| 13 | | ,24 | 69 | 77 | 67 | 29·76 | 29·79 | | 10 | 10 | SE | SE | Fine | | |
| 14 | | | 70 | 79 | 69 | 29·99 | 30·21 | | 5 | 4 | SW | W | Fine | | |
| 15 | | | 71 | 79 | 70 | 30·51 | 30·57 | | 2 | 7 | SE | SE | Fine | | |
| 16 | | | 75 | 81 | 70 | 30·42 | 30·40 | | 10 | 12 | E | E | Rain | Clo. | Fine |
| 17 | ○ | ,06 | 70 | 76 | 70 | 30·35 | 30·30 | | 7 | 9 | SE | E | Fine | | |
| 18 | | | 75 | 79 | 69 | 30·11 | 29·94 | | 10 | 9 | E | E | Fine | | |
| 19 | | | 77 | 82 | 71 | 29·90 | 29·94 | | 11 | 9 | E | NE | Fine | | |

The quantity of rain fallen in the month of June is 35-100ths only.

